# Preparation of Synthetic NiFe-Based LDH: Adsorption of Sodium Sulfadiazine from Aqueous Solution

**DOI:** 10.1021/acsomega.5c03984

**Published:** 2025-06-12

**Authors:** Elenilson Rivando dos Santos, Rayne Nunes dos Santos Martins, Yslaine Andrade de Almeida, Candice Nóbrega Carneiro, Carlos Americo Lechuga Puma, Rodrigo da Silva Viana, Iara de Fátima Gimenez

**Affiliations:** 1 Programa de Pós-graduação em Ciência e Engenharia de Materiais, 74391 Universidade Federal de Sergipe, São Cristovão, SE 49107-230, Brazil; 2 Departamento de Química, 74391 Universidade Federal de Sergipe, São Cristovão, SE 49107-230, Brazil; 3 Programa de Pós-graduação em Química, 74391 Universidade Federal de Sergipe, São Cristovão, SE 49107-230, Brazil; 4 Centro de Laboratórios de Química Multiusuários (CLQM), 74391 Universidade Federal de Sergipe, São Cristovão, SE 49107-230, Brazil; 5 Instituto de Ciências Farmacêuticas, 28112 Universidade Federal de Alagoas, Maceió, AL 57072-970, Brazil; 6 Programa de Pós-graduação em Materiais, Centro de Tecnologia, 28112 Universidade Federal de Alagoas, Maceió, AL 57072-970, Brazil

## Abstract

Layered double hydroxides (LDH) are versatile and easy-to-apply materials, and in this work, they proved to be easily synthesized using NiFe in the form of nitrates with variation of the pH in the synthesis. Its structure could be confirmed by means of X-ray diffraction, Fourier transform infrared spectroscopy (FT-IR), and transmission electron microscopy. The efficiency of NiFe-LDH as a sodium sulfadiazine (SDZ-Na) adsorbent was evaluated from isotherms and kinetics, where the data best fitted the Langmuir model, presenting a correlation coefficient of 0.9999 and a maximum adsorption capacity of 631.5 mg g^–1^, despite its moderate surface area at 117.23 m^2^ g^–1^. In addition to the adsorption capacity of NiFe-LDH, it was evident through FT-IR analysis that part of the drug used ended up adhering not only to the surface of the material but also to its layers.

## Graphical abstract

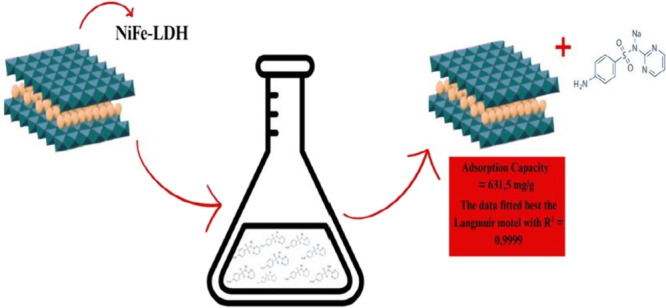

## Introduction

Environmental issues including shortcomings found in technologies for wastewater treatment are causing increasing concern,^1,2^ and among the common contaminants usually found in wastewater, a new class known as emerging pollutants (EP) has been the focus of recent research. EPs are compounds of both natural and synthetic origin that are not currently regulated but are increasingly present in water treatment plants.^3,4^ This class includes personal care and cleaning products, pharmaceuticals, industrial additives, among others whose environmental and human health effects are not fully understood.^3,5^ The amounts of pharmaceuticals detected in wastewaters near urban and industrial areas are increasing dramatically nowadays due to large production as well as uncontrolled consumption and improper disposal. This makes clear the need for the development of new purification and retention technologies to avoid dangerous consequences such as endocrine disturbances in the case of hormones and antibiotic resistance.^5–7^ The latter is a serious problem caused by the indiscriminate use of antibiotics by the human population and animal farms to promote growth and prevent bacterial infections in the latter case. Owing to their high chemical stability, antibiotics remaining in water for long times may flow through water courses contaminating also the soil, creating a whole scenario for development of drug resistance by bacterial strains, a global health threat that makes infections increasingly difficult to treat.^8^

Sulfadiazine (SDZ) is an antimicrobial drug of the sulfonamide class and is present in an acid form as well as silver (SDZ-Ag) and sodium (SZD-Na) salt forms, with the sodium salt form being highly water-soluble, in contrast to the other two. Acid form tablets are usually used to treat urinary tract infections, while sodium salt can be administered both orally and intravenously and the most common use of silver salt is topical products to treat wound infections.^9^ In this context, SZD-Na is also widely used as a broad-spectrum antibiotic to prevent infections in livestock and poultry,^10^ which causes the drug to be detected in residual waters and animal-based food.^11^ If ingested inadvertently in contaminated water and food, then SZD-Na may cause undesired effects such as allergic reactions, inhibiting enzyme activity, and imbalance of intestinal flora.^10^ SZD-Na has been often detected in urban residual waters as well as surface and subterranean natural waters contributing to the effects.^12^

Despite the situation described above, only a few reports can be found in the literature describing degradation of SZD-Na^13,14^ and adsorption of SZD-Na.^15–18^ In addition, adsorption of SZD-Na may not only lead to removal of SDZ-Na from urban and agricultural effluents but also provide a means to produce SZD-Ag in situ in membranes for wound dressing, as reported by Abid and co-workers^19^ with advantages related to the higher solubility and lower cost of SZD-Na and avoid agglomeration of SZD-Ag when used directly.

A class of anionic clays with favorable requisites to adsorb drugs in a salt form such as SDZ-Na and that remain unexplored in this context is layered double hydroxides (LDHs), considering their layered structure with positively charged layers inherent to the crystal structure and chemical composition. The structure of LDHs is formed by layers of MII­(OH)­6 octahedra in which a fraction of M^II^ cations is replaced by M^III^ ones, resulting in liquid positive charges and demanding intercalation of hydrated anions to promote electroneutrality. The general formula of LDHs is [M^II^_1–*x*_M^III^_*x*_(OH)_2_]^*x*+^A^*m*^ *x*/*m*·*n*H_2_O, where M^II^ and M^III^ represent the cations and An- is the anion present in the interlayer space.^20^ LDHs can be used in many applications including ion exchangers, polymer stabilizers, catalysts, and drug carriers and are very promising as adsorbents^21,22^ due to the relatively high surface area and adjustable chemical nature of the surface.^23,24^ Moreover, depending on the composition of LDH, i.e., the cations and anions present in the structure, guest species including molecules may be intercalated in the interlayer region through ion exchange with the original anion.^25–28^

Here, we report the preparation of LDH containing Ni^II^ and Fe^III^ as cations by coprecipitation and a study of SDZ-Na adsorption from solution including determination of adsorption isotherms and adsorption kinetics. The choice of this specific LDH to adsorb SDZ-Na was based on several criteria including the salt character of the drug, the presence of positively charged layers in the LDH, the chemical stability promoted by the presence of Fe^3+^ ions, which makes the material more stable at neutral pH for adsorption purposes,^1,24^ and previous literature data reporting the low tendency of intercalation in this LDH, thus favoring the adsorption process, in addition to the relevance of SDZ-Na adsorption.

## Experimental Section

### Chemicals

Reagents used were Fe­(NO_3_)_3_·9H_2_O (98% purity, NEON, São Paulo, Brazil), NaOH (97% purity, NEON, São Paulo, Brazil), Ni­(NO_3_)_2_·6H_2_O (98% purity, ACS Científica, São Paulo, Brazil), Na_2_CO_3_ (99.5% purity, ACS Científica, São Paulo, Brazil), and sodium sulfadiazine (C_10_H_9_N_4_NaO_2_S, 98% purity, Sigma-Aldrich Co., LLC, St. Louis, MO, USA). All reagents were used as received, and aqueous solutions were freshly prepared using deionized water from a purification system.

### Synthesis of LDH Materials

NiFe-LDH (NF) with a 3:1 Ni:Fe molar ratio was prepared by coprecipitation as follows. One hundred mL of a solution containing both Ni­(NO_3_)_2_·6H_2_O (0.3 mol L^–1^) and Fe­(NO_3_)_3_·9H_2_O (0.1 mol L^–1^) was stirred at room temperature until complete dissolution of salts. At this point, 50 mL of an aqueous solution containing both NaOH (1 mol L^–1^) and Na_2_CO_3_ (0.5 mol L^–1^) was added, and the reaction medium was stirred for 10 min. Five different pH values (8.0, 9.0, 10.0, 11.0, and 12.0) were evaluated in distinct reaction runs, adjusting the desired value through addition of NaOH. Samples were identified as NF8, NF9, NF10, NF11, and NF12 according to the pH value used. Then, the reaction mixture was placed in a Teflon-lined stainless-steel autoclave and heated at 120 °C for 24 h. After this, the solid product was filtered, washed with deionized water, and dried in an oven at 60 °C for 24 h.

### Characterization

X-ray diffraction patterns (XRD) in a 2θ range from 3 to 80° were acquired using a Shimadzu XRD-7000 (Cu Kα λ = 1.5406 Å), operating at 40 kV and 30 mA with a Ni filter and a scintillation detector, under a continuous rate mode at a 2° min^–1^ scanning rate and a step width of 0.02°. FT-IR spectra were measured from 4000 to 400 cm^–1^ using a PerkinElmer Spectrum Two instrument with KBr pellets. TG curves of samples with masses around 200 mg were acquired with a Shimadzu TG-50A from 30 to 950 °C in Pt crucibles under 100 mL min^–1^ N_2_ flow and a 10 °C min^–1^ heating rate. N_2_ adsorption/desorption isotherms were measured with a Quantachrome New 1200 instrument as follows. Samples with a mass around 100 mg were degassed during 6 h at 300 °C under vacuum prior to adsorption, and data were treated using Brunauer–Emmett–Teller (BET) to determine surface areas. Pore volumes were determined through the Dubinin–Radushkevich (DR) model, while pore diameter distributions were calculated using density functional theory (DFT). Determination of Ni and Fe was carried out by energy-dispersive X-ray spectroscopy (EDX) using a Shimadzu RayNy EDX-720 by applying voltages from 15 to 50 kV and using a 100 μA current value, a 40% dead time, and a collimator of 10 mm. Samples were irradiated for 100 s under vacuum, and spectra were sequentially acquired at the 0–40 keV energy range. Determinations of C, H, and N (CHN analysis) were carried out for samples weighing around 30 mg using a LECO CHN628 instrument operating with helium gas (99.995% purity) and oxygen (99.999%), calibrated with an EDTA standard (41% C, 5.5% H, and 9.5% N), heating the samples at 950 °C in the furnace, with an afterburner temperature of 850 °C. Morphological evaluation was carried out by scanning electron microscopy (SEM) with a JSM-5700 JEOL microscope at 10–20 kV and by transmission electron microscopy (TEM) with a JEOL JEM 1400 Plus operating at 120 kV.

### Adsorption Isotherms

Experiments were performed by adding 1 g of LDH (sample NF9 was selected owing to characteristics explained in the Discussion) to 100 mL of SDZ-Na (C_10_H_9_N_4_NaO_2_S) solutions with concentrations ranging from 100 to 5000 mg L^–1^, followed by stirring at room temperature during 24 h. Then, the solids were separated by filtration, washed with deionized water, and dried in an oven at 60 °C for 24 h, while the concentration of SDZ-Na in the liquid phase was determined by UV/visible spectrophotometry (Shimadzu UV-1800 instrument) by measuring the absorbance at 263 nm. The adsorption capacity values (*q* in mg g^–1^) were calculated using eq 1:

$$q=\frac{V\left(C_{0}-C_{e}\right)}{m}$$ 1

where *C*_0_ and *C*_e_ are the initial and equilibrium SDZ-Na concentrations (mg L^–1^), *V* is the volume of the solution (L), and *m* is the adsorbent mass (g).

### Kinetic Study

A mass/*V* (L) ratio of 1 g L^–1^ of LDH was employed in the kinetic study using 5000 mg L^–1^ SDZ-Na solutions under constant stirring at room temperature. Aliquots were withdrawn from the suspensions at predefined times (1–2700 min) and filtered to separate the solid, and the absorbance at 263 nm of the liquid phase was determined using a UV-1800 Shimadzu instrument.

## Results and Discussion

### Preparation of NiFe-LDH

Samples of NiFe-LDH obtained at different pH values showed predominance of the LDH phase for all major peaks, which agree with the crystallographic record ICSD-81963.^29,30^ Figure 1 presents the XRD patterns obtained, evidencing characteristic peaks of LDH at 2θ = 11.5, 23.0, 34.4, 38.8, 60.1, and 61.2°, confirming the formation of the layered structure typical of these materials.

**Figure 1:**
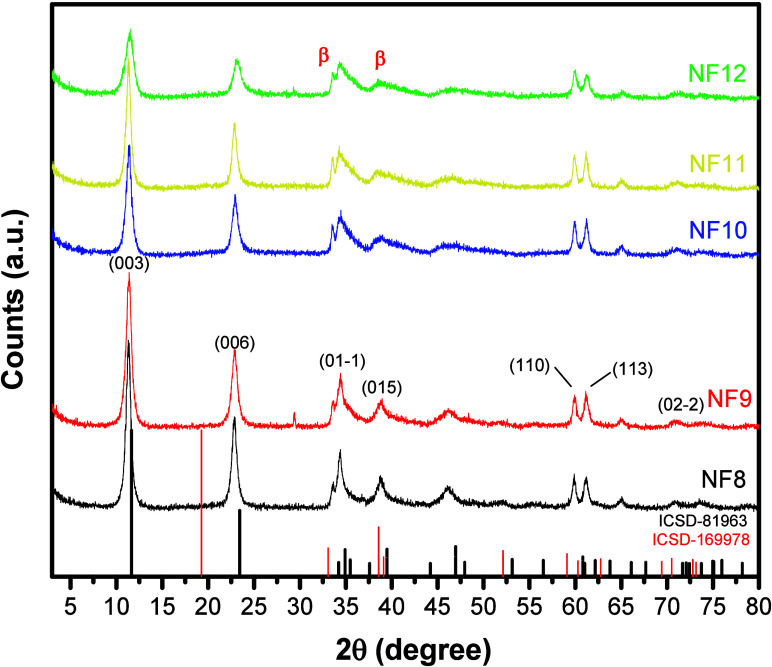
XRD patterns of samples NF8, NF9, NF10, NF11, and NF12 prepared via a coprecipitation reaction, with phase identification based on crystallographic reference files ICSD-81963 (LDH phase) and ICSD-169978 (β-Ni­(OH)_2_ phase).

However, it was observed that the increase in pH led to the formation of the β-Ni­(OH)_2_ phase (ICSD-169978),^31^ reflected by the broadening and decrease in the intensity of the signals, which suggests a reduction in the crystallinity of specific planes of the LDH. The low crystallinity observed may be related to the disordered growth of the lamellar layers or the presence of structural impurities, resulting in lower crystalline organization.^32^ In addition, sample NF12 is more evident at 2θ = 33.3 and 38.8°, which are attributed to this β-Ni­(OH)_2_ phase.^31^ The coexistence of these two phases suggests that variations in the pH during synthesis significantly influenced the purity and structural organization of the material.

FT-IR spectra (Figure 2) evidence further differences among the samples precipitated under different pH values, as NF10, NF11, and NF12 samples exhibited a band at 1005 cm^–1^ with intensity upon an increase in pH, which is absent for NF8 and NF9 samples. This band is assigned to Ni–O stretching from Ni­(OH)_2_, and its behavior indicated that above pH 9, the precipitation process does not lead to a pure LDH phase, as reported in the literature for other LDHs.^31^ Spectra for all samples show bands characteristic of LDH materials such as the band at 3054 cm^–1^ assigned to OH stretching from both structural hydroxyls and water molecules^29,33^ and the band at 1642 cm^–1^ due to angular deformation of water molecules. The band at 3001 cm^–1^ is assigned to OH stretching of hydroxyl groups interacting with intercalated anions,^29^ while the bands at 1370, 970, and 750 cm^–1^ are assigned to asymmetric modes of carbonate ion^34,35^ and the band at 1384 cm^–1^ is due to the ν_3_ vibration from nitrate.^35–38^ Thus, FT-IR spectra show that LDH materials prepared here contain mixtures of carbonate and nitrate ions in the interlayer regions, which is consistent with the use of nitrates as precursor salts and the common presence of carbonate resulting from CO_2_ dissolved in water. As both NF8 and NF9 were found to be free of Ni­(OH)_2_, sample NF9 was selected for further characterization and application tests due to the higher precipitation yield during the synthesis.

**Figure 2:**
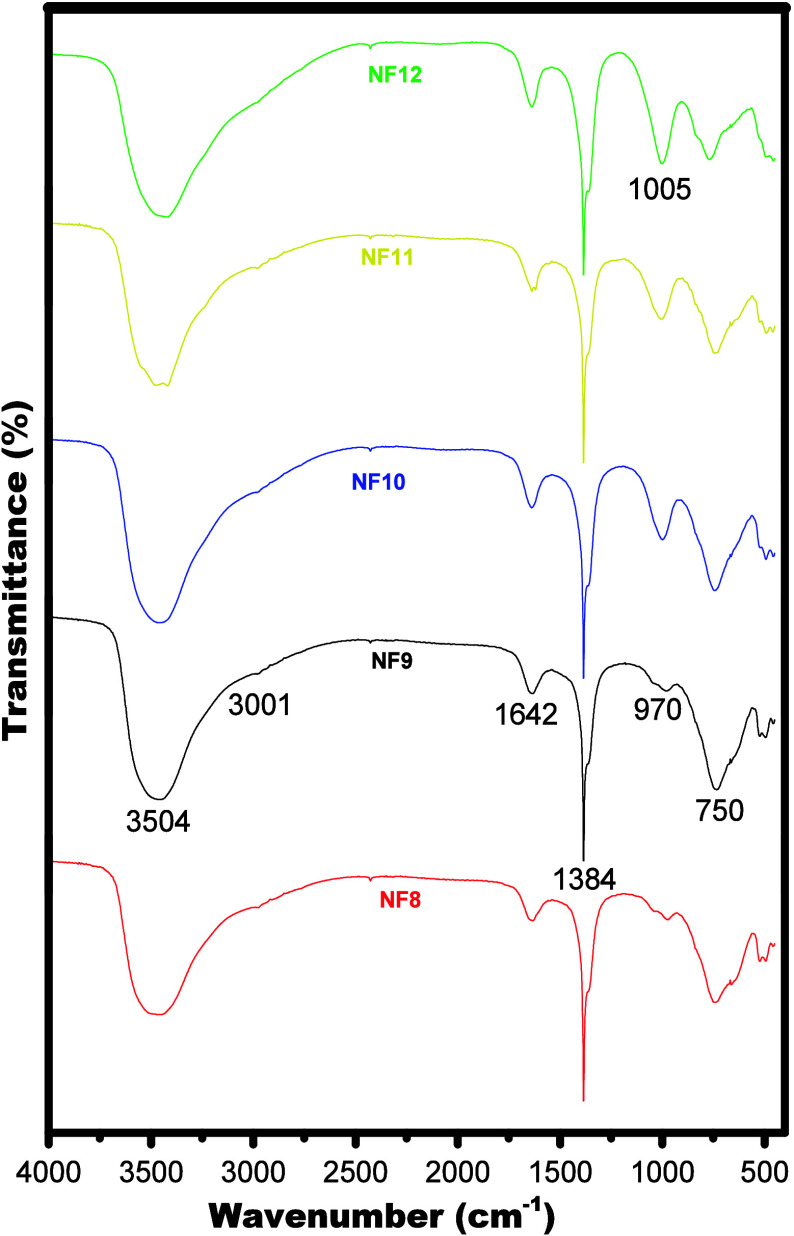
FT-IR spectra of samples NF8, NF9, NF10, NF11, and NF12 prepared via a coprecipitation reaction.

TG analysis of the NF9 LDH sample (Figure 3) shows distinctive mass loss processes, including a weight loss of 2.48% at 48 °C due to physically adsorbed water molecules, followed by other losses at 164 °C (9.45%) with release of interlayer water molecules and at 269 °C (13.43%) assigned to dehydroxylation of the lamellae possibly combined with carbonate degradation.^39^ Finally, an additional event occurred at 446.11 °C, which was not reported for NF containing only carbonate ions,^40^ which may be tentatively assigned to nitrate decomposition.

**Figure 3:**
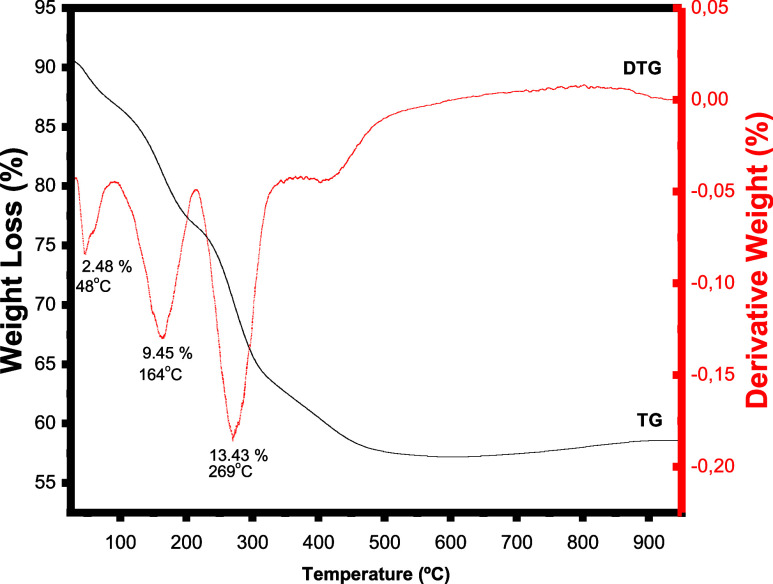
TG/DTG curves of sample NF9.

Figure 4a shows the isotherms obtained, whose behavior corresponds to type IV­(a), according to the classification of the International Union of Pure and Applied Chemistry (IUPAC),^41^ characterized by the presence of type H3 hysteresis. The measured surface area was approximately 117.23 m^2^ g^–1^ (±0.14), while the pore volume was 0.086 cm^3^ g^–1^ (±0.19). This isotherm reflects the presence of mesopores throughout the pressure range but also of possible nanopores due to the concentration of the volume variation in diameters smaller than 2.0 nm. According to the pore size distribution curve (Figure 4b), the material exhibits pores mainly in the range of *X* to *Y* nm, with an average pore diameter estimated at approximately 1.62 nm based on the density functional theory (DFT) model. However, the low intensity values on the *Y*-axis indicate a limited pore volume, suggesting that the material has a relatively low accessible porosity. This behavior is also consistent with the adsorption/desorption isotherms (Figure 4A), which show a moderate adsorbed volume. Therefore, although the material presents certain microporous or mesoporous features, its overall porosity is limited.

**Figure 4:**
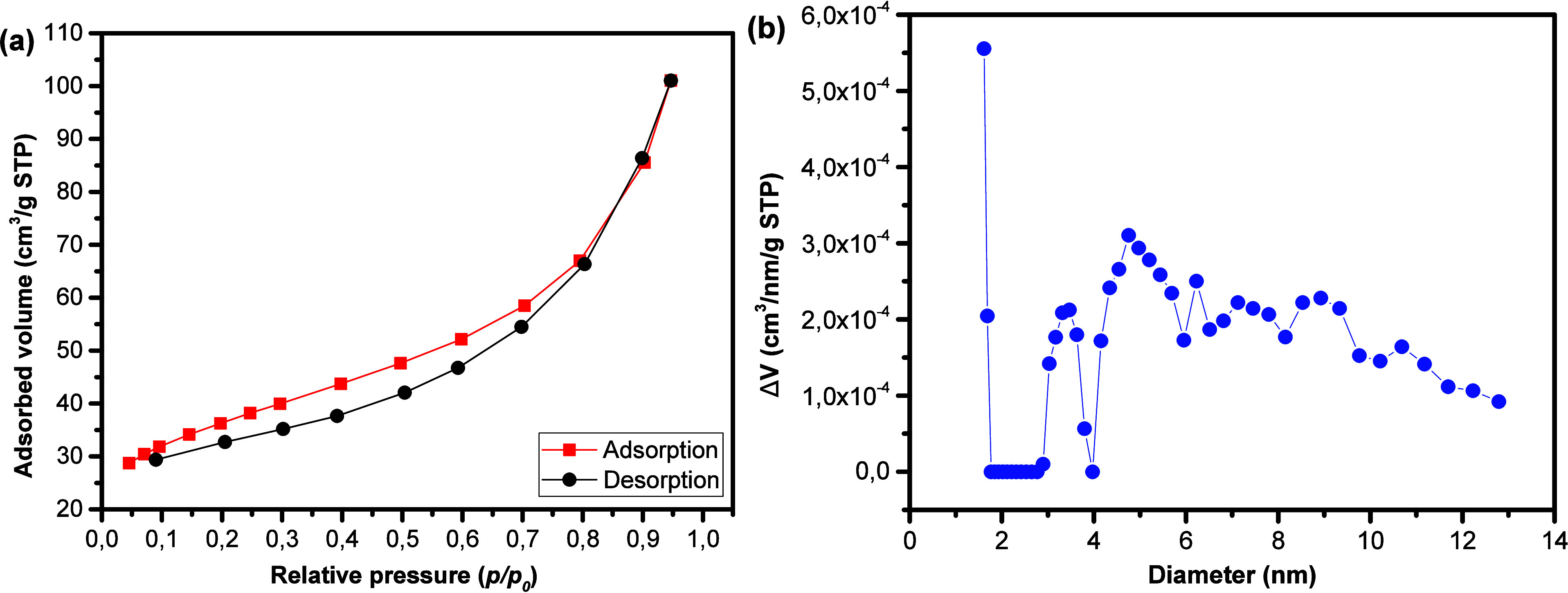
N_2_ adsorption/desorption isotherms (a) and DFT pore diameter distribution of the NF9 sample (b).

The morphology of sample NF9 as observed by SEM microscopy (Figure 5a) is characterized by the presence of irregular particles probably formed by the aggregated primary particles, where it is possible to verify only the surface of HDL as well as its topography. Moreover, the plate-like morphology typical of LDHs can be distinguished under TEM observation (Figure 5b, c, e, and f). The plates present in the samples tend to be hexagonal in shape^42–44^ with sizes around 45 nm (Figure 5b) in the direction parallel to the surface, based on measurements carried out using ImageJ software.

**Figure 5:**
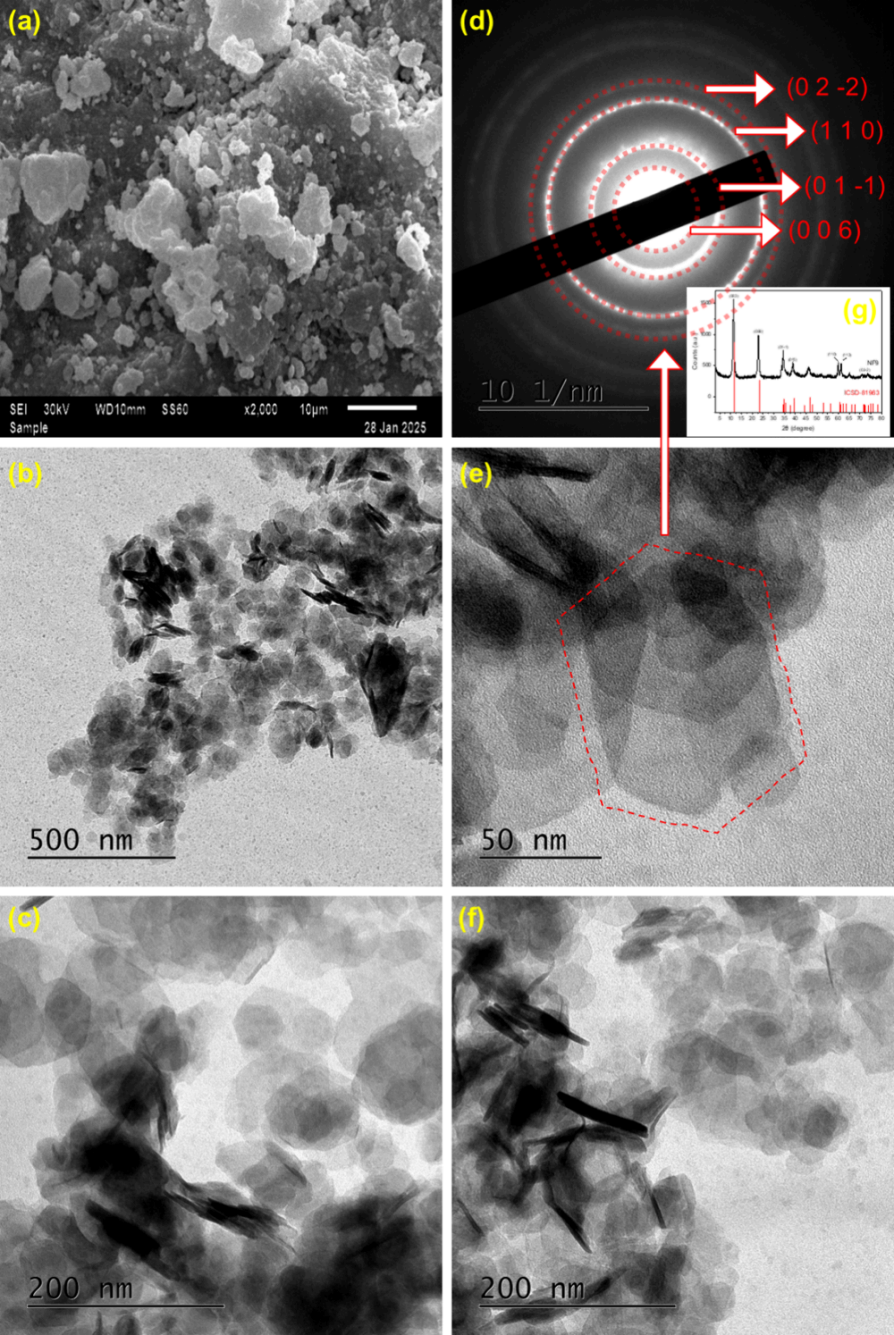
SEM image at 2.000× magnification (a); TEM micrographs at 12.000× (b), 40.000× (c, f), and 100.000× (e) magnifications; SAED pattern (d); XRD pattern (g) of the NF9 sample.

From Figure 5e, it was possible to determine the interplanar distances by analyzing the SAED pattern (Figure 5d), using CrysTBox software for crystallographic visualization and automated electron diffraction analysis.^45^ The image reveals well-defined diffraction rings and based on the crystallographic record ICSD-81963, interplanar distances (*d*-spacing) of 0.481, 0.264, 0.155, and 0.132 nm were identified, corresponding to the crystallographic planes (006), (011̅), (110), and (022̅) of the NiFe-LDH. These results agree with the diffraction peaks previously identified by XRD, confirming a good structural ordering and possibly indicating the formation of a single NiFe-LDH phase and similar to other studies reported in the literature.^46,47^

Elemental analyses by CHN and EDX are presented in Table 1 and confirmed the presence of 1.42% (±0.04) of nitrogen probably from nitrate ions, in agreement with the addition of this ion in the synthesis and FT-IR/TG results. In addition, EDX data revealed that the Ni:Fe proportion was 3.6:1 showing that a lower proportional Fe^3+^ was incorporated into the structure compared with the nominal composition, which is not uncommon for synthetic LDH materials and reflects differences in the valence and size of the bivalent and trivalent cations.

**Table 1:** Chemical Composition of the NF9 Sample and Molar Ratio from the Synthesis

sample	Ni/Fe ratio	%Ni (w/w)	%Fe (w/w)	%C (w/w)	%H (w/w)	%N (w/w)
NF9	3.60	79.09 ± 0.27	20.91 ± 0.33	1.37 ± 0.09	2.95 ± 0.13	1.42 ± 0.04

### Adsorption of SDZ-Na with NiFe-LDH

Considering the existence of positive charges in the LDH layers and the anionic character of SDZ-Na, here, we propose an evaluation of the adsorption from a solution of SDZ-Na onto LDH based on the determination of adsorption isotherms and a kinetic study. The two isotherm models studied here were Langmuir and Freundlich models, represented in eqs 2 and 3 in nonlinear forms:

$$\mathrm{Langmuir}:\;q_{e}=\frac{q_{\max}K_{L}C_{\mathrm{eq}}}{1+K_{L}C_{\mathrm{eq}}}$$ 2

where C_eq_ (mg L^–1^) is the equilibrium concentration of the adsorbate in the liquid phase after adsorption, *q*_e_ (mg g^–1^) is the equilibrium amount adsorbed in mass (mg) of adsorbate per mass (g) of adsorbent, *q*_max_ is the estimation of maximum adsorptive capacity from the Langmuir isotherm, and *K*_L_ and *K*_F_ are the Langmuir and Freundlich constants.

$$\mathrm{Freundlich}:\;q_{e}=K_{F}C_{e}^{n}$$ 3

Figure 6 shows the plots with experimental points as well as the curves obtained by fitting the data to both models. The values of the coefficient of determination (*R* ^2^) resulting from the fitting processes and the parameters characteristic of both models are shown in Table 2. Both models are well-fitted, but the Langmuir model was found to be slightly better with an *R* ^2^ value of 0.9999, which indicated that the adsorption process takes place with formation of a monolayer without interaction between adsorbate molecules.

**Figure 6:**
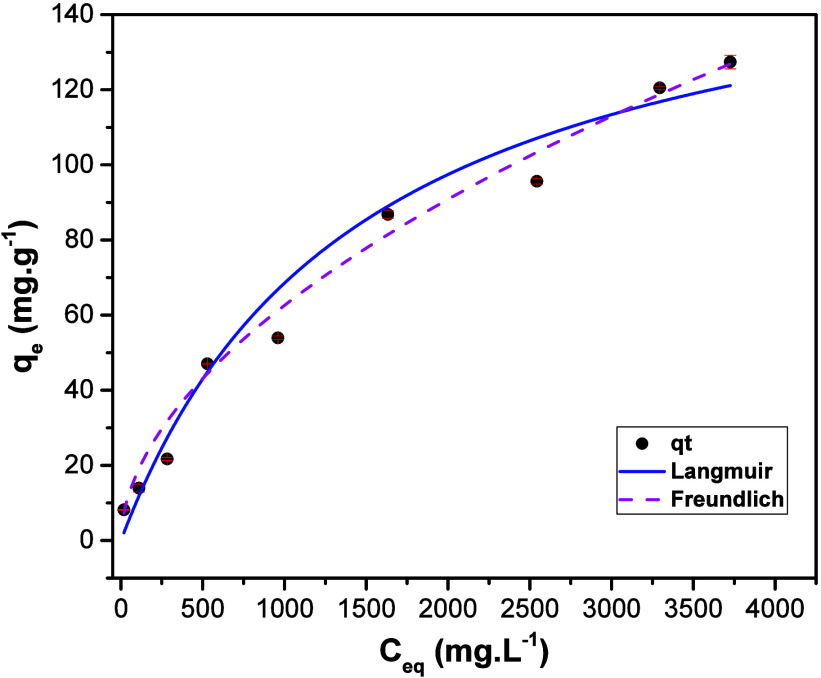
Langmuir and Freundlich adsorption models for SDZ-Na adsorption on NiFe-LDH.

**Table 2:** Langmuir and Freundlich Parameters for SDZ-Na Removal by NiFe-LDH

Langmuir			Freundlich		
*R* ^2^	*Q*_max_ (mg g^–1^)	*K*_L_ (L mg^–1^)	*R* ^2^	*K*_F_ ((mg g^–1^)(L mg^–1^)^1/*N*^)	*N*_F_
0.9999	631.5 ± 0.76	1.07 × 10^–3^	0.9849	1.0286	0.5863

Compared to the few literature works reporting adsorbents for SDZ-Na (Table 3), the maximum adsorptive capacity found here can be considered very good particularly considering the moderate value of the surface area. Usually, high capacities are exhibited by biochars, with surface areas above 2000 m^2^ g^–1^.

**Table 3:** Comparison of *Q*_max_ with Other Adsorbents Found in the Literature for SDZ-Na

material	surface area (m^2^ g^–1^)	*Q*_max_ (mg g^–1^)	refs.
ionic liquid-functionalized polystyrene		431.034	^15^
molecularly imprinted spheroidal carbonized polymer dots	204	61	^17^
	219	9.54	^17^
biochar	2343	950	^16^
NiFe-LDH	117.2 (±0.14)	631.5 (±0.76)	this work

Although the surface area of the NF used for this adsorption was moderate when compared to other materials reported in the literature, it is important to emphasize that during the aging of an LDH in a solution containing dissociated cations, other mechanisms of interaction between the material and the drug may be favored. In the case of the NF reported here, the main reason for this superior performance was due to the high availability of active sites present on its surface, which contributes to the generation of electrostatic bonds between the SDZ-Na cations, enriching the positive charges already present in the LDH layers and, therefore, its interaction with the SDZ-Na anions increases the adsorption capacity.^1^

Furthermore, the other mechanism linked to this adsorption capacity at 631.5 mg g^–1^ (±0.76) occurs due to the anion exchange capacity of the material’s lamellae, which allows SDZ-Na to be exchanged for the NO_3_^–^ anions present in the material’s lamellae initially and thus the drug to be removed from the aqueous medium.^24^

After adsorption, four LDH samples were selected for characterization by FT-IR (Figure 7a) and XRD (Figure 7b). The samples were renamed NFX*x*, where the second *x* represents the concentration of SDZ-Na used in the adsorbed solution (NFX5, NFX10, NFX25, and NFX50). The FT-IR spectra changed significantly, mainly with changes in the region of vibrations of the anion present in the interlayer region. No clear peaks from SDZ-Na appear in the spectra probably owing to a relatively low concentration, particularly peaks around 1550 cm^–1^ attributed to phenolic structures linked to NH_2_ groups, in addition to peaks at 1240 and 630 cm^–1^ that can be attributed to asymmetric S=O stretching. Regarding peaks from LDH, the sharp peak at 1385 cm^–1^ assigned to the ν_3_ vibration from nitrate is significantly reduced for the NFX5 sample and the shoulder at 1355 cm^–1^, assigned to carbonate, turns into a relatively broad band, which remains in the spectra of all samples. This is followed by the subtle appearance of SDZ-Na bands marked with arrows in the spectrum of NFX50.

**Figure 7:**
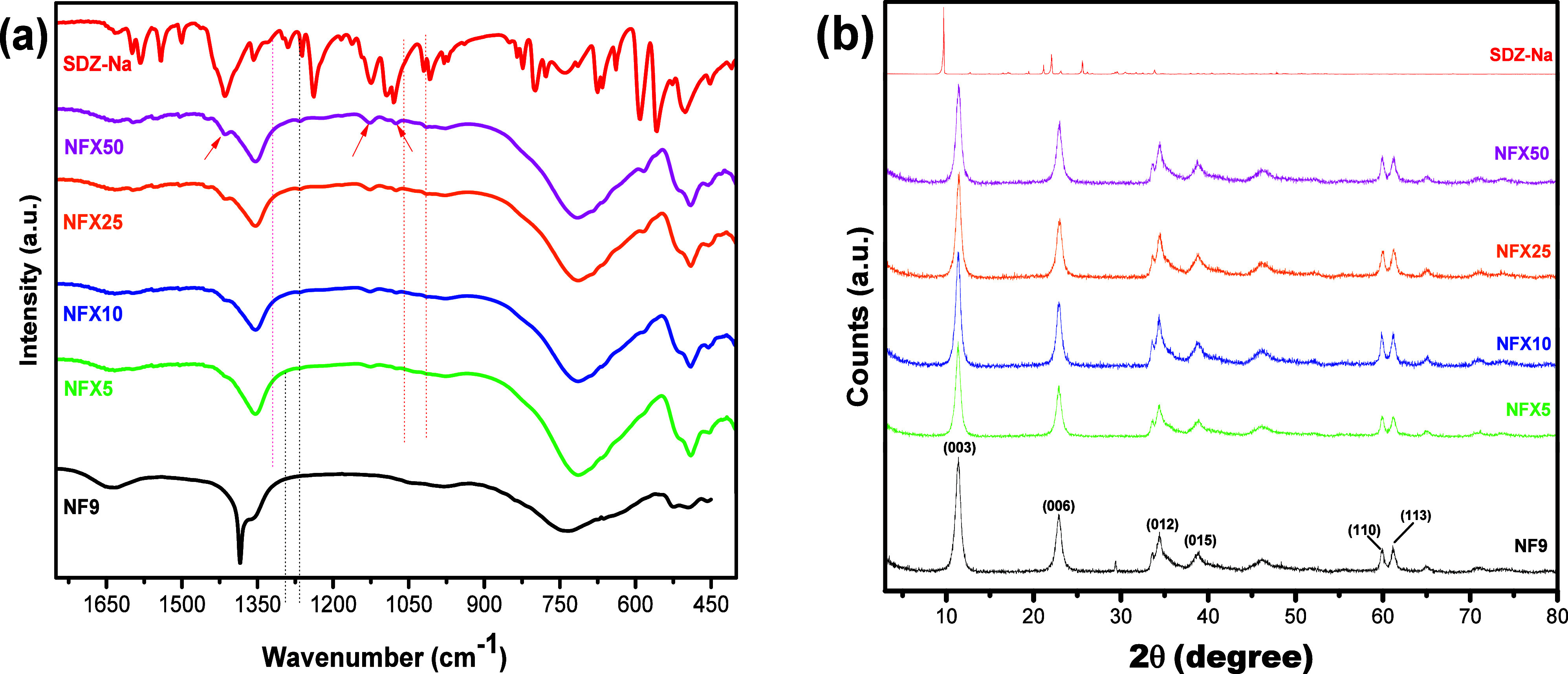
FT-IR spectra (a) and XRD patterns (b) of NF9 after adsorption in different concentrations of the SDZ-Na solution.

Observations from FT-IR are consistent to partial intercalation of SDZ-Na by exchange with NO_3_^–^ and originally present in the interlayer region of LDH, as an analogous behavior of anion bands was reported following intercalation of methotrexatum in MgAl LDH.^48^ Here, XRD patterns do not show any shift of the basal peak (003) to lower angles as usually expected to occur after intercalation of species larger than original anions. Regarding situations when XRD analysis is not conclusive to distinguish between intercalation and surface adsorption, Varga and co-workers^49^ studied differences in FT-IR spectra when organic compounds are intercalated or adsorbed onto the surface of CaAl LDHs. This was carried out using different IR techniques (photoacoustic, PAS), attenuated total reflection (ATR), diffuse reflection (DRIFTS), and wide-angle diffuse reflection (WA-DRIFTS) with different penetration depth profiles, showing that the removal of part of the original anions concomitantly with the presence of bands of intercalated molecules evidences partial intercalation. Thus, a combined analysis of the changes in FT-IR and in XRD patterns suggests that nitrate ions were exchanged by SDZ molecules here while carbonate remained intercalated. Moreover, adsorption via intercalation explains the relatively high adsorption capacity of the LDH in view of its moderate surface area. Intercalation does not occur in an expressive manner since it is possible that the anion exchange of carbonate ions cannot be identified by FT-IR; however, it is possible to suggest a partial intercalation of SDZ-Na molecules, mainly because it was possible to identify the decrease in the presence of nitrate in the material lamellae, which also suggests that NO_3_^–^ molecules are more susceptible to anion exchange during intercalation using a NiFe system as LDH.^24^

To verify the thermal behavior of the sample that best adsorbed the drug, TG curves for SDZ-Na and NFX50 are shown in Figure 8 (a and b, respectively). The TG curve for SDZ-Na (Figure 8a) shows a sharp event with a peak in the DSC curve at 397 °C related to drug decomposition, which is absent in the TG curve obtained for NFX50, probably due to the low drug amount. The TG curve of NFX-50 (Figure 8b) is similar to that of the free LDH sample, while the DTG curve showed that the event related to dehydroxylation shifted from 269 to 295 °C, evidencing that the presence of SDZ-Na molecules in the interlayer region changed the energies involved in this particular event.

**Figure 8:**
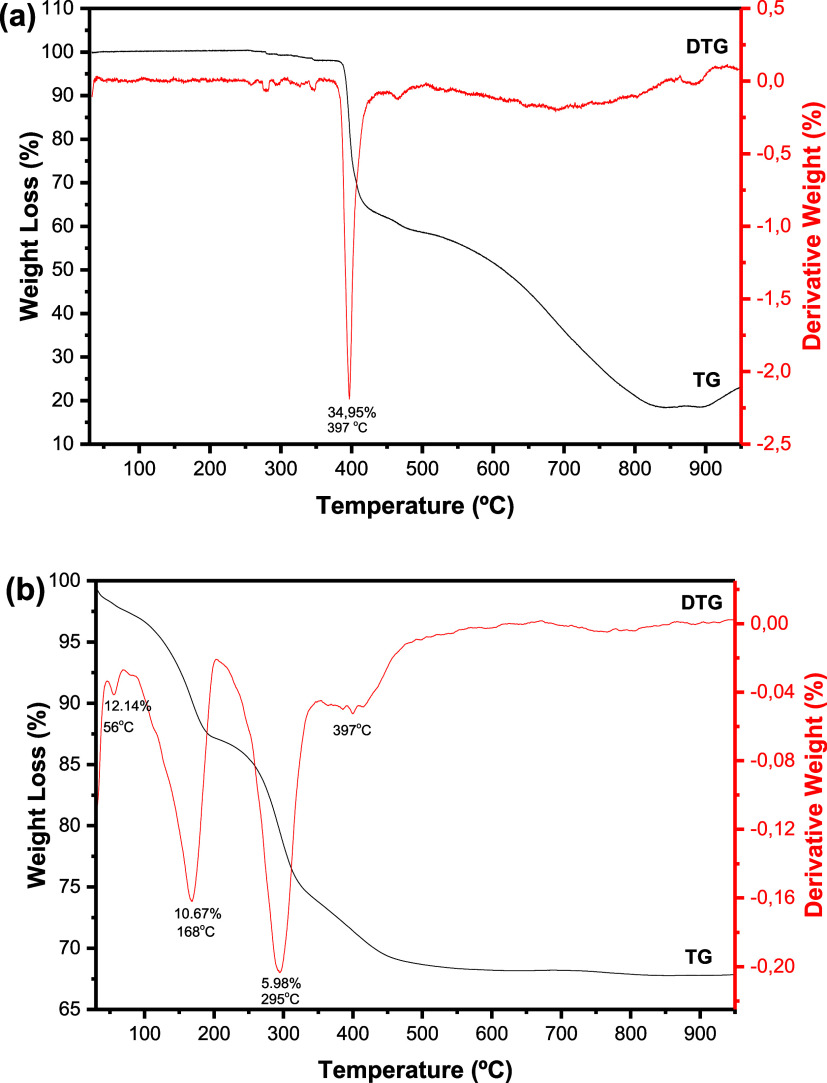
TG/DTG curves for SDZ-Na (a) and NFX50 (b).

### Contact Time Effect on SDZ-Na Adsorption

The effect of contact time can be observed in Figure 9, revealing that the adsorption is very fast in the first 5 min and assumes a different kinetic regime after 10 min, starting to exhibit distinctive steps, a common occurrence for pharmacological species.^50,51^

**Figure 9:**
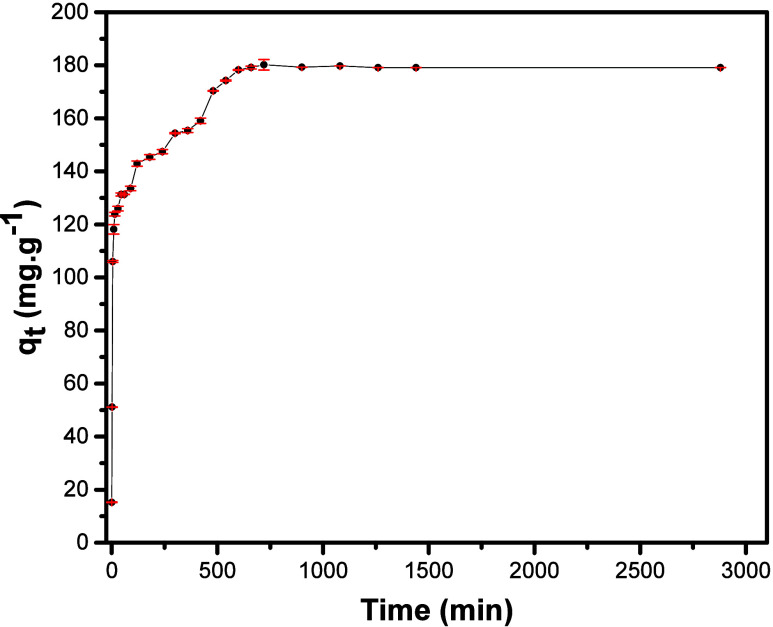
Contact time effect of the adsorption for SDZ-Na in the NF9 sample.

For this test, as the process occurs, it is possible to verify that the adsorptive capacity reaches a plateau after 900 min since the concentration becomes constant, indicating a *q*_e_ of 179.27 mg g^–1^. The experimental data were used to test different kinetic models in linear forms, including pseudo-first-order (PFO, eq 4), pseudo-second-order (PSO, eq 5), and Weber and Morris’s intraparticle diffusion models (eq 6):

$$\ln\left(q_{e}-q\right)=\ln\left(q_{e}\right)-k_{1}\times t$$ 4

where *q*_e_ is the removal percentage, *t* is the time (min), and *k*_1_ is the pseudo-first-order constant (min^–1^).

$$\frac{1}{q}=\frac{1}{k_{2}\times q_{e}^{2}}+\frac{1}{q_{e}}\times t$$ 5

where *q_t_* and *q*_e_ are the adsorption capacities as a function of time and at equilibrium, respectively, in mg g^–1^, and *k*_2_ (h^–1^ (mg g^–1^) is the pseudo-second-rate constant.

$$q=k_{i}\times t^{0.5}$$ 6

where *q* is the adsorption capacity (mg g^–1^) at time *t* (min) and *k*_i_ is the intraparticle diffusion rate constant (mg g^–1^ h^–0.5^).

The kinetic parameters obtained applying the models (Figure 10) can be found in Table 4 and provide evidence that PSO (Figure 10b) is the best model to describe data obtained here, with an *R* ^2^ of 0.9981. According to the literature, this model is usually appropriate to fit data related to relatively short adsorption times or when the adsorbent–adsorbate interactions take place fast^52^ being the reason for this behavior for the adsorption of SDZ-Na on NF (Figure 9). In this case, a series of factors may have influenced the initial adsorption rate to be fast, such as the surface area of NF available for adsorption, the ease of the hydroxide layers in adsorbing molecules that undergo speciation in a neutral form, such as SDZ-Na,^11,49^ and also the anion exchange property of LDH, which may favor partial intercalation in the material’s lamellae.

**Figure 10:**
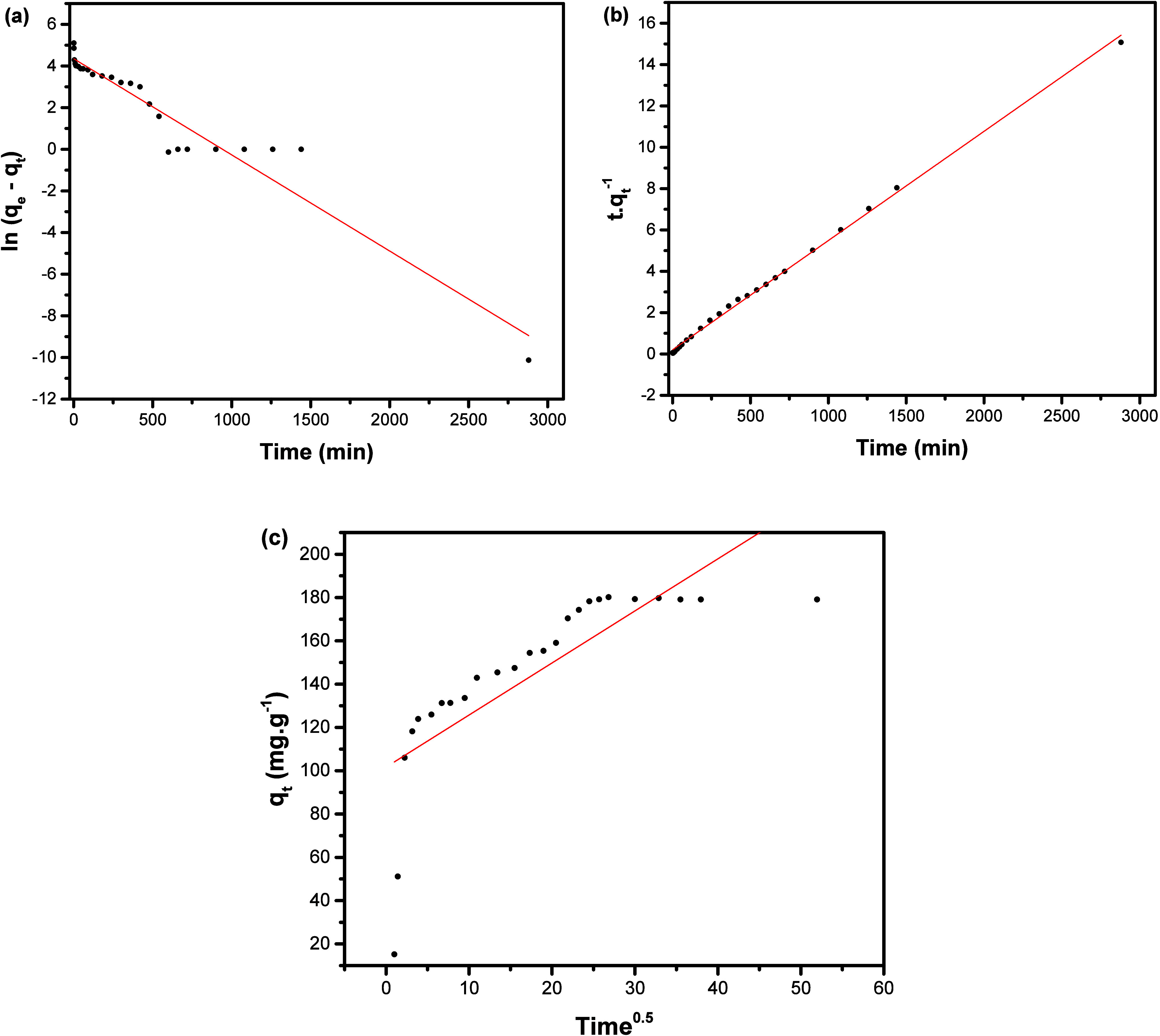
Linear plots of pseudo-first-order (a), pseudo-second-order (b), and intraparticle diffusion (c) kinetic reaction isotherms of SDZ-Na adsorption by NiFe-LDH.

**Table 4:** Kinetic Parameters for SDZ-Na Adsorption by NiFe-LDH

model	parameter	NF9 sample
pseudo-first order	*q*_e_ (mg g^–1^)	81.6915
	*K*_1_ (min^–1^)	–0.2347
	*R* ^2^	0.9136
pseudo-second order	*q*_e_ (mg g^–1^)	25.6937
	*K*_2_ (min^–1^ (mg g^–1^))	0.00742
	*R* ^2^	0.9981
intraparticle diffusion	*K*_i_ (mg g^–1^ min^0.5^)	9.0064
	*R* ^2^	0.6031

The performance of the other models (Figure 10a, FSO, and Figure 10c for the intraparticle diffusion) tested was found to be poor, so their specific mechanistic assumptions can be ruled out.

Adsorption processes, especially when using LDHs as adsorbents, mostly combine chemical and physical adsorption since this occurs in several stages.^43^ It is possible that SDZ-Na molecules can undergo diffusion in the NF structure through the mesopores and the surface area of the material itself, generating anion exchange for cations that are already present in the NF lamellae, in this case NO_3_^–^ and CO_3_^2–^, since the surface area of the LDH is positively charged.^48,53^ In this sense, it is not possible to clearly determine the main adsorption mechanism used in this study solely by using and evaluating the three kinetic models, only that adsorption with SDZ-Na on the surface of the NF occurs as in other studies that use drugs as adsorbates.^52^

Furthermore, as *q_t_* increases rapidly in the initial minutes according to the PSO model, it is possible that part of the subsequent equilibrium occurs due to anion exchange promoted by a partial replacement of the nitrate ions that are allocated in the NF lamellae, which also explains the mg g^–1^ ratio and may be a suggestion of partial intercalation.^25,49^

## Conclusions

In this work, layered double hydroxides based on iron and nickel nitrate salts were successfully prepared by the coprecipitation method, varying the pH of the synthesis between 8.0 and 12.0 and subsequent heat treatment at 120 °C for 24 h. The material synthesized at pH 9.0 was the one that obtained the highest degree of crystallinity and therefore was used as a new adsorbent for the removal of sodium sulfadiazine in aqueous solution. This study indicates that the sorption step of SDZ-Na with NF9 follows the Langmuir isotherm model, presenting a correlation coefficient of 0.9999 for the material, and therefore, it is believed that the drug is formed on the surface of the LDH in a monolayer system.

Furthermore, the adsorption kinetics best fitted the pseudo-second-order model, and the maximum adsorption capacity until equilibrium was 179.27 mg g^–1^. Thus, the results discussed not only present the characterization of a route for the preparation of NF but also demonstrate important information that allows the evaluation of the development of new technologies for the treatment of wastewater that contains minimum doses of drug in its composition, leaving as a suggestion for future work the comparative evaluation of the adsorbent after calcination and in systems with new adsorbates of the same class used.

In addition, during the interaction of SDZ-Na with NF, it is possible to note that this does not occur only through adsorption in the surface area but also a possible partial intercalation of the drug in the layers of the material, which was evidenced through the FT-IR analysis and due to the good adsorption capacity of the material, although it is characterized by having a moderate surface area, which corroborates the understanding that the prepared material also has good ion exchange capacity.
